# Hypervirulent *Acinetobacter baumannii* (hvAB): The Convergence of Virulence and Multidrug Resistance

**DOI:** 10.3390/antibiotics14060551

**Published:** 2025-05-29

**Authors:** Nan Wu, Xinqian Ma, Wentao Ni

**Affiliations:** Department of Respiratory and Critical Care Medicine, Peking University People’s Hospital, Beijing 100044, China

**Keywords:** *Acinetobacter baumannii*, multidrug resistance, hypervirulent

## Abstract

*Acinetobacter baumannii* has become a formidable pathogen in healthcare systems worldwide, primarily due to its remarkable capacity to develop multidrug resistance and cause life-threatening infections. While traditionally *A. baumannii* is considered an opportunistic pathogen of low virulence, accumulating evidence now underscores the emergence of hypervirulent *A. baumannii* (hvAB) strains. These strains combine heightened pathogenicity with extensive drug resistance, posing unprecedented challenges for clinical management and infection containment. This review comprehensively explores the molecular mechanisms driving hvAB’s virulence and antimicrobial resistance and its evolutionary trajectory, clinical presentations, and global epidemiology. Additionally, we evaluate potential therapeutic strategies and their broader public health implications.

## 1. Introduction

*Acinetobacter baumannii*, a Gram-negative, non-fermentative opportunistic pathogen, has emerged as a leading cause of healthcare-associated infections (HAIs) globally [1,2,3]. This pathogen thrives in hospital environments due to its exceptional capacity to persist on abiotic surfaces, resist desiccation, and tolerate common disinfectants [2,4]. A study of 100 *A. baumannii* isolates from an intensive care unit (ICU) showed that all strains had the ability to form biofilms, and this ability was clearly linked to their environmental adaptability [5]. Immunocompromised patients and those with invasive medical devices in the ICUs are at highest risk. *A. baumannii* can cause a variety of severe infections such as ventilator-associated pneumonia (VAP), bloodstream infections (BSIs), surgical site infections, and urinary tract infections (UTIs) [4,6,7,8].

Antimicrobial resistance (AMR) is a defining feature of *A. baumannii* and a major contributor to its growing clinical impact [3,9]. The global spread of multidrug-resistant (MDR) and extensively drug-resistant (XDR) strains has become particularly concerning [10,11]. A systemic review of 126 studies across 29 countries revealed that MDR strains caused 79.9% of hospital-acquired pneumonia (HAP) and VAP cases [12]. These strains exhibit resistance to a wide range of antibiotics, including carbapenems, fluoroquinolones, aminoglycosides, and even polymyxins and tigecycline, which have been treated as last-resort agents due to their broad-spectrum antibacterial activity [4,10]. Key resistance mechanisms involve carbapenemase production (e.g., OXA-23, NDM-1, and IMP), outer membrane protein modifications (e.g., CarO), and efflux pump overexpression [13]. The multidimensional nature of resistance has earned *A. baumannii* designation as an “critical priority” pathogen on the WHO’s list of bacteria needing immediate action for the development of novel therapeutic strategies [3,6,10,14].

Despite its complex resistance profile, *A. baumannii* has been traditionally regarded as a low-virulence pathogen primarily affecting immunocompromised hosts [4,7,15]. However, recent evidence highlights the rise of hypervirulent strains that pose new clinical challenges [10]. These strains exhibit enhanced pathogenicity, including increased tissue invasiveness and improved immune evasion capabilities when infecting immunocompetent individuals [7]. Although hvAB strains remain relatively uncommon, their combination with MDR raises concerns about the potential for more severe, untreatable infections. Understanding the mechanisms underlying this convergence of resistance and virulence is essential in guiding future therapeutic and infection control strategies. This review synthesizes the current knowledge on *A. baumannii*, examining its virulence factors, AMR mechanisms, clinical impacts, evolutionary pathways, and epidemiology trends, with special emphasis on the emerging threat of hypervirulence.

## 2. Virulence Factors of *A. baumannii*

The host innate immune system, particularly neutrophils and macrophages, serves as the primary defense against *A. baumannii* infection [16]. *A. baumannii* was previously considered a low-virulence pathogen; however, emerging evidence reveals that specific strains possess enhanced pathogenic capabilities [4]. For example, the bacterial enzyme RecA mediates DNA repair and resistance to desiccation, enabling *A. baumannii* intracellular survival within macrophages [17,18]. These virulence determinants collectively promote environmental persistence and immune evasion, and the ability to infect both immunocompromised and immunocompetent hosts. While the definition of hypervirulence continues to evolve, elucidating these fundamental virulence mechanisms remains essential for understanding *A. baumannii* pathogenicity (Table 1).

### 2.1. Capsule and Hypermucoviscosity

The polysaccharide capsule, present in nearly all *A. baumannii* strains, is a key virulence factor that facilitates immune evasion [2,3]. Studies have demonstrated that the capsule protects the bacterium from phagocytosis and complement-mediated killing, allowing it to persist and proliferate in the host [2]. Multiple capsule-encoding genes are involved in the process of capsule formation. Mutations in these related genes can lead to changes in the bacterial capsule function, resulting in immune evasion and increased virulence of the bacteria [3]. The primary genetic determinant of capsule production is the K locus. The size of the K locus can vary between 20 and 35 kB, reflecting the genetic diversity of the complex sugar-synthesis region. To date, 237 distinct K loci have been identified [2,14]. For example, research has proved that KL49 is an independent predictors of mortality [19]. Another gene is *grt6*, classified as ISAba13, belonging to Insertion Family 5 and Group 903, which allows bacteria to easily attach to immune cells, be engulfed, and remain non-lethal in the living body [3]. The *grt6* mutant exhibits reduced phagocytosis in vitro and impaired clearance in vivo, leading to lethal infections [3].

Beyond immune evasion, the capsule promotes bacterial adhesion, biofilm formation, and cell-surface interactions, contributing to hypermucoviscosity—a phenotype linked to enhanced survival, resistance to neutrophil killing, and tissue invasion. This trait has been observed in hvAB strains [14], though its clinical significance remains less defined compared with other pathogens (e.g., *Klebsiella pneumoniae*) [20]. Regulatory systems, such as the BfmRS two-component system, tightly control capsule synthesis, and mutations in these pathways may concurrently affect virulence and antibiotic susceptibility [21].

### 2.2. Iron Acquisition Systems

Iron is an essential trace element for both humans and bacterial pathogens, and plays a central role in numerous cellular processes. In humans, the majority of iron is sequestered within cells or bound to proteins, leaving only minimal free iron available in serum. However, bacteria require higher concentrations of iron to survive [22]. Therefore, to overcome this limitation in the iron-restricted host environment, *A. baumannii* has evolved sophisticated iron acquisition systems, including siderophores (acinetobactin, fimsbactins, and baumannoferrin), hemophore-mediated heme uptake, and ferrous-iron transporters [23].

Among these, the siderophore-mediated systems are particularly important for virulence, and have been identified as one of the effective strategies by which bacteria evade host nutritional immunity [23]. The biosynthesis and transport of acinetobactin, encoded by the *bas* and *bau* operons, are upregulated during infection and allow the bacterium to scavenge iron from host proteins such as transferrin and lactoferrin [23]. Additionally, the heme utilization system, encoded by the *hemO* cluster, another contributor to virulence, facilitates the acquisition of iron from heme, providing a critical nutrient source during infections [8,24]. Notably, hypervirulent strains frequently exhibit enhanced expression of these iron acquisition systems, granting them a competitive advantage in iron-deprived host environments and promoting rapid proliferation during infection.

### 2.3. Outer Membrane Proteins (OMPs) and Secretion Systems

OMPs are integral components of the bacterial outer membrane and play pivotal roles in host–pathogen interactions. As key constituents of outer membrane vesicles (OMVs), OMPs mediate critical functions such as nutrient/drug transport, bacterial adhesion, and immune evasion [25]. Among these, *ompA*—the most abundant porin in *A. baumannii*—stands out for its multifunctional contributions to virulence. It facilitates host tissue adhesion, biofilm formation, and immune evasion [25,26]. The binding of *ompA* to host fibronectin initiates bacterial attachment to host cells [27,28,29]. Once internalized via OMVs, *ompA* localizes to mitochondria, triggering cytochrome c and apoptosis-inducing factor (AIF) release, ultimately inducing epithelial cell apoptosis [30,31].

In addition to OMPs, *A. baumannii* utilizes secretion systems to enhance virulence. The Type VI secretion system (T6SS) is a recently identified virulence factor that contributes to bacterial competition, host cell invasion, and immune modulation [4]. By delivering effector proteins into competing microbes or host cells, T6SS enhances bacterial survival in diverse niches. While the role of T6SS in hypervirulence is not yet fully elucidated, its activity is strongly associated with bacterial fitness and adaptability [32]. Additionally, the Type II secretion system (T2SS) secretes γ-glutamyltransferase (GGT), an enzyme associated with exacerbated infection severity [33].

### 2.4. Biofilm Formation

Biofilm formation is one of *A. baumannii*’s most well-documented virulence traits. Meanwhile, biofilm formation serves as a protective mechanism against antibiotics. Bacteria within biofilms exhibit antibiotic resistance up to 1000-fold greater than their planktonic counterparts [22]. Notably, *A. baumannii* demonstrates superior biofilm-forming capacity compared with other Acinetobacter species [34], providing critical survival advantages against desiccation, antibiotics, and host immune defenses [14].

In *A. baumannii*, the biofilm-associated protein (*bap*) plays a central role in biofilm development, promoting surface adhesion and intercellular aggregation [14]. Other regulators, such as quorum-sensing systems (*abaI/abaR*), modulate biofilm architecture and density in response to environmental signals [10]. Studies have shown that inactivation of the *abaI* mutation can lead to a reduction of up to 40% in biofilm formation in *A. baumannii* [10,35]. Hypervirulent strains often exhibit enhanced biofilm production, which acts as a physical barrier, reducing antibiotic penetration and promoting persistence in healthcare environments. Within biofilms, horizontal gene transfer (HGT) is facilitated, accelerating the acquisition and dissemination of resistance determinants. This convergence of virulence and resistance mechanisms renders hvAB infections particularly recalcitrant to treatment.

## 3. AMR Mechanisms

The resistance mechanisms in *A. baumannii* can be broadly categorized into enzymatic degradation, target modification, efflux pump overexpression, and reduced membrane permeability [36] (Table 2):

### 3.1. Enzymatic Degradation

*A. baumannii*’s resistance to β-lactam antibiotics is primarily mediated by the production of β-lactamases, which degrade the β-lactam ring of the antibiotics, rendering them ineffective [37]. Based on the Ambler classification system, the β-lactamases are classified into four types [38]:

Class A (serine-β-lactamases): These β-lactamases catalyze the hydrolysis of the β-lactam ring using a serine residue at the active site, with common examples including penicillinases and cephalosporinases. Their genes can be easily transferred from one organism to another through HGT [37,39].

Class B (metallo-β-lactamases, MBLs): This type of enzyme is the only β-lactamase that uses metal ions as a catalyst. Enzymes such as NDM-1 (New Delhi metallo-β-lactamase), IMP (imipenemase), and VIM (Verona integron-encoded metallo-β-lactamase) hydrolyze a broad spectrum of β-lactams, including carbapenems [36,37,39]. MBLs are typically encoded on mobile genetic elements (MGEs), contributing to their rapid spread in clinical isolates [36].

Class C (AmpC β-lactamases): This type of enzyme is intrinsic to all *A. baumannii* [36]. It is an important cephalosporinase, mediating resistance to most penicillins, cephalosporins, β-lactamase inhibitors, and β-lactam combinations [40]. Upregulation of the expression of this gene is one of the main mechanisms by which *A. baumannii* develops resistance to cephalosporins [41].

Class D (OXA-type β-lactamases): These include OXA-23, OXA-24/40, OXA-58-like, OXA-143, and OXA-51-like enzymes, which are frequently associated with carbapenem resistance [10,42]. Among them, OXA-51-like is an intrinsic gene, while the other genes are acquirable [42]. OXA-23 is particularly widespread globally and is often encoded on plasmids or transposons, spreading through HGT via MGEs [10,43].

### 3.2. Target-Site Modifications

Resistance to fluoroquinolones, such as ciprofloxacin and levofloxacin, is mediated by mutations in DNA gyrase (*gyrA*) and topoisomerase IV (*parC*), which reduce drug binding. Similarly, aminoglycoside resistance is achieved through methylation of the 16S ribosomal RNA, mediated by enzymes like ArmA [44]. Resistance to polymyxins (e.g., colistin) is typically mediated by modifications to the lipopolysaccharide (LPS) structure, reducing the binding affinity of polymyxins. Mutations in genes such as *pmrA*, *pmrB*, and *lpxD* are responsible for these alterations [45,46].

### 3.3. Efflux Pumps

Efflux pumps actively expel antibiotics from the bacterial cell, reducing intracellular drug concentrations, enabling survival, and inducing antibiotic resistance adaptation [47]. The four common efflux pumps of *A. baumannii* include the resistance-nodulation-division (RND) family, the multidrug and toxic compound extrusion (MATE) family, the major facilitator superfamily (MFS), and the small multidrug resistance (SMR) family [13].

Among these, RND-type pumps are the most clinically significant in MDR strains [48]. The AdeABC efflux pump, which are often widely overexpressed in MDR A. baumannii, confers resistance to aminoglycosides, tigecycline, and fluoroquinolones, and plays a dual role in hvAB strains [42]. Overexpression is often driven by mutations in regulatory genes such as *adeR* and *adeS* [42]. The AdeIJK pump also mediates intrinsic resistance to multiple antibiotic classes [49], and the AdeFGH pump works synergistically with the AdeIJK pump to enhance broad-spectrum resistance [49].

Notably, efflux pumps also play an important role in hypervirulence. The murine infection model shows that the *acrB* (AcrAB efflux pump encoding gene) knockout *K. pneumoniae* strain has a reduced capacity to cause pneumonia, suggesting that AcrAB efflux pump is involved in pathogenicity [50]. Atin Sharma et al. proved that the AdeF pump participates in biofilm formation, and C. elegans worms feeding on the *abaF*-mutant had a longer survival time, suggested that the AbaF pump is involved in the virulence formation of *A. baumannii* [51].

### 3.4. Outer Membrane Porin Modifications

Reduced membrane permeability to prevent the entry of antimicrobial agents into the bacterial cell is another critical resistance mechanism [36,52]. *A. baumannii* achieves this by modifying or losing porins, which are proteins that facilitate the uptake of antibiotics [52]. Of particular importance is the CarO porin, which plays an essential role in carbapenem penetration [53]. Mutations, insertions, or deletions in the *carO* gene reduce antibiotic influx, thereby conferring resistance to carbapenems [52].

## 4. Evolutionary Pathways

The emergence of hvAB strains reflects a complex evolutionary process driven by the convergence of virulence and AMR mechanisms [54,55]. While virulence and resistance were historically viewed as distinct traits, increasing evidence has demonstrated their coexistence in clinical isolates [55,56]. Unlike classical MDR strains, hvAB integrates genetic traits that simultaneously enhance both pathogenicity and resistance, resulting in highly adaptable and clinically formidable strains. This integration takes place through multiple mechanisms, including HGT, co-evolution of virulence and resistance genes, and adaptive mutations. MGE and genomic plasticity also play a core role in the process [54].

### 4.1. Horizontal Gene Transfer

HGT serves as a primary mechanism for antimicrobial resistance acquisition in hvAB [14]. The histone-like nucleoid structuring protein (H-NS) plays a critical regulatory role in this process, where its absence leads to significant overexpression of seven HGT-related genes (*pilA*, *pilT*, *pilQ*, *comEA*, *comEC*, *comF*, and *drpA*) [57]. The efficiency of HGT in *A. baumannii* is largely mediated by MGEs, including plasmids, transposons (Tn), integrons, and insertion sequences (ISs) [57,58,59], which has led to its recognition as a highly proficient organism for natural gene exchange and evolution [60]. These elements can simultaneously carry both virulence factors—such as those involved in capsule biosynthesis or siderophore production—and resistance genes—such as carbapenemases (OXA-23 and NDM-1), extended-spectrum β-lactamases (ESBLs), or colistin resistance genes (e.g., *mcr-1*) [13]. Furthermore, outer membrane vesicles (OMVs) enhance HGT by packaging and transferring genetic material, including resistance genes, between bacterial cells. OMVs are especially significant in the context of hvAB, as they also carry virulence-associated factors, enhancing the pathogenic potential of recipient strains. This remarkable genomic plasticity enables *A. baumannii* to rapidly adapt to diverse environmental pressures, including antimicrobial therapies and host immune defenses.

#### 4.1.1. Acquisition of Virulence Plasmids by MDR Strains

MDR *A. baumannii* strains can acquire virulence plasmids carrying genes that encode critical virulence factors, including capsule biosynthesis loci (KL loci), siderophore production operons (*bas/bau* operons), and secretion systems (e.g., T6SS) [61]. For example, some MDR strains enhance their ability to acquire iron in iron-limited environments by acquiring plasmids that carry genes encoding siderophore biosynthesis systems, enabling the strains to proliferate in iron-limited environments, and facilitating infection [61].

#### 4.1.2. Acquisition of Resistance Plasmids by Hypervirulent Strains

hvAB strains, which are inherently more pathogenic due to their virulence factors, can acquire resistance plasmids to become MDR hvAB. These plasmids often carry resistance genes such as carbapenemases (OXA-23, NDM-1) [34]. This plasmid acquisition confers robust antibiotic resistance to otherwise virulent strains, significantly enhancing their survival capacity in clinical settings, and complicating treatment options.

#### 4.1.3. Integration of Resistance and Virulence Genes into Composite Plasmids

The formation of composite plasmids containing bothAMR and virulence determinants represents the most sophisticated evolutionary adaptation in hvAB. These hybrid genetic elements co-localize critical genes, including those encoding carbapenemases and efflux pumps (AMR) alongside capsule synthesis and siderophore production systems (virulence), ensuring their coordinated expression and stable co-inheritance.

Insertion sequences, such as ISAba1, play a pivotal role in this process by driving genetic rearrangements and promoting the integration of resistance and virulence genes into composite plasmids [49]. For instance, IISAba1-mediated enhancement of carbapenemase gene expression and dissemination has been well documented. These composite plasmids not only maintain stable resistance–virulence linkages but also exhibit increased horizontal transfer efficiency, significantly accelerating the spread of hvAB.

### 4.2. Co-Evolution of Virulence and Resistance

The interplay between AMR and virulence in *A. baumannii* is increasingly attributed to the shared regulatory systems that simultaneously mediate bacterial survival under antibiotic pressure and enhance pathogenicity. These systems, such as two-component systems (TCSs) and quorum-sensing mechanisms, provide dual benefits by balancing fitness, resistance, and virulence in diverse environments [62].

For instance, the PmrAB system modifies the lipid A component of lipopolysaccharides (LPSs), which reduces colistin binding (AMR) while evading host immune recognition (virulence) [55]. Similarly, the BfmRS system coordinately regulates both capsule production and biofilm formation, providing dual advantages: the capsule protects against host immune defenses (virulence) while biofilms create a physical barrier against antimicrobial agents (AMR) [62]. These examples demonstrate how shared regulatory mechanisms intrinsically connect resistance and virulence traits, enabling *A. baumannii* survival under challenging environmental conditions.

Quorum sensing (*abaI/abaR*) reinforces this connection by simultaneously regulating biofilm formation and virulence-factor expression while indirectly contributing to antibiotic resistance through the formation of protective microenvironments for bacterial persistence [10]. Additionally, the AdeRS TCS regulates efflux pumps, such as AdeABC, which expel antibiotics and enhance bacterial survival under stress, thereby contributing to both resistance and host adaptation. Iron acquisition systems, crucial for bacterial survival in iron-limited host environments, often co-localize with resistance genes on plasmids. These genetic associations, coupled with selective pressures from antibiotic exposure and host immune defenses, have driven the emergence of strains combining multidrug resistance with hypervirulence.

### 4.3. Adaptive Mutagenesis

The dual pressures of antibiotic exposure and host immune responses can induce mutagenic adaptations in *A. baumannii*, which concurrently enhance both antimicrobial resistance and virulence properties [62]. For example, mutations in the BfmRS regulatory system coordinately modulate capsule biosynthesis to improve immune evasion while simultaneously affecting antibiotic susceptibility profiles [21,63,64]. Adaptive genomic changes facilitate the emergence of hvAB variants with optimized survival capabilities in both clinical settings and host microenvironments.

## 5. Clinical Impacts

*A. baumannii* colonizes environmental surfaces and medical devices, posing a significant threat through severe healthcare-associated infections (HAIs), especially in immunocompromised patients or those with prolonged hospitalization [10,54,65]. While MDR *A. baumannii* is a well-established nosocomial pathogen, the emergence of hvAB strains has introduced a new dimension of clinical concern [10,66]. These strains, which combine high virulence with MDR, are associated with more severe infections and worse clinical outcomes.

### 5.1. Infections and Disease Severity

hvAB has been reported to cause a variety of serious infections, including VAP, BSI, meningitis, UTI, and surgical site infections [7,54,66,67]. These infections caused by hvAB are often more severe than those caused by classical *A. baumannii*, and they are increasingly being reported in both immunocompromised and immunocompetent patients [54,68].

#### 5.1.1. Ventilator-Associated Pneumonia

*A. baumannii* is a leading cause of VAP in ICUs, particularly in patients requiring prolonged mechanical ventilation [2,61]. A single-center clinical study demonstrated that hvAB-associated VAP led to significantly higher rates of bacteremia and mortality compared with non-hypervirulent strains [69].

#### 5.1.2. Bloodstream Infections

A single-center study including 31 cases of BSI found that hv-CRAB strains significantly increased septic shock risk [7]. Key virulence factors for BSI include capsular polysaccharides, T6SS, and biofilm formation [70,71,72].

#### 5.1.3. Meningitis

hvAB causes severe postsurgical meningitis in neurosurgical patients, often exhibiting treatment resistance and poor outcomes [6]. in vitro studies showed that cerebrospinal fluid (4%) upregulates *A. baumannii* virulence factors (pili, fimbriae, and siderophores) and may interact with human serum albumin [6], potentially enhancing iron acquisition via *bas/bau* gene cluster activation [15].

### 5.2. Mortality and Treatment Strategies

Infections caused by hvAB demonstrate significantly higher mortality rates compared with classical MDR *A. baumannii* infections [8], with reported mortality rates reaching 50–70% for hvAB bloodstream infections [7]. Survivors frequently experience severe complications including prolonged hospitalization, secondary infections, and long-term organ dysfunction. The clinical impact is further exacerbated by limited effective treatment options for MDR hvAB. While colistin and tigecycline serve as last-resort agents, their efficacy is often compromised by resistance mechanisms including *pmrA/B* mutations and efflux pump overexpression [73]. Clinicians are therefore constrained to combination therapies such as colistin–tigecycline or carbapenem-based regimens, though these approaches demonstrate suboptimal therapeutic outcomes.

Emerging antimicrobial agents show promise for MDR hvAB treatment. Cefiderocol (Shionogi & Co., Ltd., Osaka, Japan) exhibits potent activity against Ambler class A–D β-lactamases and demonstrates superior or non-inferior efficacy compared with colistin and tigecycline against MDR *A. baumannii* [74]. Elavacycline, a novel fluorocycline antibiotic, displays broad-spectrum activity against MDR Gram-negative, Gram-positive, and anaerobic pathogens, with in vitro studies showing 2–8-fold lower MIC values against MDR *A. baumannii* compared with tigecycline [75]. The recent FDA approval of sulbactam/durlobactam (May 2023) represents another therapeutic advance, with the multinational ATTACK trial demonstrating non-inferiority to polymyxins in 28-day all-cause mortality for carbapenem-resistant *A. baumannii* infections, including MDR strains [76,77]. However, current efficacy data for these novel agents primarily derive from studies of MDR *A. baumannii*, and their clinical performance against MDR hvAB requires further investigation.

## 6. Surveillance and Control Measures

Surveillance data reveal regional variations in MDR prevalence in *A. baumannii*: 77–87% in Africa, Asia, and Latin America; 47% in North America; and exceeding 93% in the Middle East and Europe [54,78]. Studies have revealed that hvAB strains are becoming increasingly prevalent in those regions, particularly in Asia, the Middle East, and Europe [7]. The global emergence of hvAB strains, combined with high multidrug resistance prevalence, poses a significant public health threat.

Robust surveillance is critical for monitoring the emergence and spread of hvAB strains. Whole genome sequencing (WGS) and molecular typing techniques, such as multi-locus sequence typing (MLST), are essential for tracking clonal lineages and identifying resistance–virulence linkages [8].

Additionally, improved infection control measures—such as hand hygiene, environmental disinfection, and antimicrobial stewardship—are vital for limiting the spread of hvAB in healthcare settings. Addressing hvAB spread demands a coordinated global response, with key priorities including the following:

1. Enhancing diagnostic capabilities for early hvAB detection.
2. Developing robust genomic surveillance systems to track circulating strains.
3. Fostering international cooperation through a unified global tracking platform for monitoring cross-border transmission.

## 7. Conclusions

The emergence of hvAB is becoming a significant challenge due to its combination of enhanced virulence and MDR, causing severe infections such as VAP, BSI, and meningitis, often with high morbidity and mortality. The evolutionary trajectory of hvAB is propelled by HGT, genomic plasticity, and regulatory systems (e.g., PmrAB and BfmRS) that functionally link AMR and virulence. Molecular epidemiology data underscore the global spread of hvAB, particularly in Asia, the Middle East, and Europe. Addressing this threat requires enhanced surveillance, strengthened infection control, and the development of pathogen-specific therapeutics. Future research priorities must elucidate hvAB’s pathogenesis, resistance mechanisms, and transmission patterns. A coordinated global response is not only essential but urgently needed to prevent hvAB from following the dangerous trajectory of hypervirulent *K. pneumoniae*, which has caused devastating worldwide outbreaks. To effectively combat hvAB, we urgently need to establish a global surveillance network while intensifying research into both its virulence mechanisms and potential anti-virulence strategies.

## Figures and Tables

**Table 1:** Functions of virulence factors.

Gene	Function	Virulence Mechanism
**Capsule and Hypermucoviscosity**		
K locus	Capsule basic coding gene	
*grt6*	Promote phagocytosis in vivo	Mutant strain: innate immune escape
BfmRS system	Capsule synthesis	Mutant strain: influence virulence and antibiotic susceptibly
**Iron Acquisition Systems**		
Siderophore-mediated systems	Evade host nutritional immunity	Allow bacteria to obtain nutrients from the host
*bas/bau*	Promote the scavenging of iron from host proteins	Provide nutrition for bacteria
*hemO*	Promote acquisition of iron from hemoglobin	Provide nutrition for bacteria
**Outer Membrane Proteins and Secretion Systems**		
*ompA*	Facilitate adhesion to host tissues, promote biofilm formation, and promote apoptosis in epithelial cells	Immune evasion and epithelial cell apoptosis
T6SS	Bacterial competition, host cell invasion, and immune modulation	Provide survival advantages in external and host environments
T2SS	Secrete GGT	Cause more severe infection
**Biofilm Formation**		
*bap*	Promote surface adhesion and intercellular aggregation	Essential in biofilm development
*abaI/abaR*	Modulate biofilm architecture and density	Modulate biofilm in response to environmental signals

**Table 2:** Key resistance mechanisms in Acinetobacter baumannii.

Enzymes/Gene	Characteristic	Target Antibiotics
**Enzymatic degradation**		
Class A (serine-β-lactamases)		
Penicillinases and cephalosporinases	Catalyze the hydrolysis of the β-lactam ring	Penicillin, cephalosporin, carbapenem
Class B (metallo-β-lactamases)		
NDM-1/IMP/VIM enzymes	Use metal ions as catalysts, typically encoded on MGEs	Carbapenems
Class C (AmpC β-lactamases)		
Cephalosporinase	Mediating resistance to penicillin, cephalosporin, β-lactamase inhibitor, and β-lactam combination	Cephalosporins
Class D (OXA-type β-lactamases)		
OXA-23, OXA-24/40, OXA-58-like, OXA-143 and OXA-51-like enzymes	OXA-51-like is innate, the others are acquirable	Carbapenems
**Target Site Modifications**		
DNA gyrase (*gyrA*), topoisomerase IV (*parC*)	Reduce drug binding	Fluoroquinolones
ArmA enzyme	Methylation of the 16S ribosomal RNA	Aminoglycoside
*pmrA, pmrB, lpxD*	Modifications to LPS structure, reduce binding affinity	Polymyxins
**Efflux Pumps**		
RND family		
AdeABC efflux pump (*adeR, adeS*)	Function both with antibiotics and hypervirulence	Aminoglycosides, tigecycline, fluoroquinolones
AdeIJK and AdeFGH pump	Main cause of intrinsic resistance and cause of broad-spectrum resistance	
**Outer Membrane Porin Modifications**		
CarO	Essential for carbapenem entry	Carbapenems (after variation)

## Data Availability

Not applicable.

## Abbreviations

The following abbreviations are used in this manuscript:

AMR	Antimicrobial resistance
Bap	Biofilm-associated protein
BSI	Bloodstream infection
CRAB	Carbapenem-resistant *A. baumannii*
ESBL	Extended-spectrum β-lactamase
GGT	γ-glutamyltransferase enzyme
HAI	Healthcare-associated infection
HGT	Horizontal gene transfer
H-NS	Histone-like nucleoid structuring protein
hvAB	Hypervirulent *A. baumannii*
ICU	Intensive care unit
IS	Insertion sequence
LPS	Lipopolysaccharide
MDR	Multidrug resistance
MGE	Mobile genetic elements
MLST	Multi-locus sequence typing
OMP	Outer membrane protein
OMV	Outer membrane vesicle
T2SS	Type II secretion system
T6SS	Type VI secretion system
TCS	Two-component system
UTI	Urinary tract infection
VAP	Ventilator-associated pneumonia
WGS	Whole-genome sequencing
WHO	World Health Organization
XDR	Extensively drug-resistant
